# Functional relationship between p53 and RUNX proteins

**DOI:** 10.1093/jmcb/mjy076

**Published:** 2018-12-11

**Authors:** Suk-Chul Bae, Arun Mouli Kolinjivadi, Yoshiaki Ito

**Affiliations:** 1 Department of Biochemistry, School of Medicine, and Institute for Tumour Research, Chungbuk National University, Cheongju, South Korea; 2 Cancer Science Institute of Singapore, National University of Singapore, Singapore, Singapore

**Keywords:** RUNX, tumour suppressor, p53

## Abstract

RUNX genes belong to a three-membered family of transcription factors, which are well established as master regulators of development. Of them, aberrations in RUNX3 expression are frequently observed in human malignancies primarily due to epigenetic silencing, which is often overlooked. At the G1 phase of the cell cycle, RUNX3 regulates the restriction (R)-point, a mechanism that decides cell cycle entry. Deregulation at the R-point or loss of RUNX3 results in premature entry into S phase, leading to a proliferative advantage. Inactivation of Runx1 and Runx2 induce immortalization of mouse embryo fibroblast. As a consequence, RUNX loss induces pre-cancerous lesions independent of oncogene activation. p53 is the most extensively studied tumour suppressor. p53 plays an important role to prevent tumour progression but not tumour initiation. Therefore, upon oncogene activation, early inactivation of RUNX genes and subsequent mutation of p53 appear to result in tumour initiation and progression. Recently, transcription-independent DNA repairing roles of RUNX3 and p53 are emerging. Being evolutionarily old genes, it appears that the primordial function of p53 is to protect genome integrity, a function that likely extends to the RUNX gene as well. In this review, we examine the mechanism and sequence of actions of these tumour suppressors in detail.

## Introduction

Cancer develops primarily due to loss of tumour suppressive activity and gain of oncogenic activity. Transcription factor RUNX1 is a master player of haematopoiesis, RUNX2 is indispensable for osteoblast development, and RUNX3 is an essential factor for the development of T cell and other multiple tissues. All three RUNX proteins exert dual functions: oncogenic and tumour suppressive, depending on the cellular context (Blyth et al., 2005, 2010; Kilbey et al., 2007, 2008). We therefore describe RUNX proteins as Jekyll and Hyde (Ito et al., 2015). RUNX is regulated by various developmental regulator signals, such as Wnt, Hedghog, and TGF-β. Furthermore, RUNX3 is frequently downregulated epigenetically in various types of cancer, including gastric, breast, lung, pancreas, prostate, oesophagus, and uterine cervix cancer (Lee et al., 2017). p53, in the presence of DNA damage or strong oncogenic stimuli, transactivates p21 to inhibit DNA synthesis and induce cell cycle arrest or apoptosis (el-Deiry et al., 1993; Waga et al., 1994). Here, we present mechanistic insights into how RUNX3 and p53 act independently and in concert to elicit their tumour suppressive function. Finally, we summarize recent findings on transcription-independent DNA repair function of RUNX and p53 genes.

## Tumour suppressive functions of p53 and RUNX

### p53 restoration prevents adenocarcinoma but not adenoma in mouse models

It has been demonstrated that ectopic expression of oncogenic K-*Ras* in normal cells induces apoptosis rather than cellular transformation and that the p14ARF–p53 pathway plays key roles in the defence against aberrant oncogene activation (Levine, 1997; Serrano et al., 1997; Kruse and Gu, 2009). Simultaneous activation of oncogenic K*-Ras* and inactivation of the *p53* tumour suppressor in mouse lung significantly accelerate malignant progression to adenocarcinoma (DuPage et al., 2009). Considering the pro-apoptotic function of p53, restoration of p53 appears to be an attractive therapeutic intervention. Two independent groups tested this hypothesis in a mouse model of K-*ras*^*G12D*^ lung adenocarcinoma (Feldser et al., 2010; Junttila et al., 2010). Interestingly, both studies concluded that p53 restoration resulted in a significant decrease of adenocarcinoma, with cells displaying features of senescence-like cell cycle arrest. Within 3 days of p53 restoration, p21, the p53 target gene, was readily detectable at the sites of adenocarcinoma (Feldser et al., 2010). As a consequence of p21 accumulation, the percentage of tumours within adenocarcinoma was significantly reduced within 2 weeks of p53 restoration. However, in marked contrast, p53 restoration did not eliminate adenoma cells as efficiently as adenocarcinomatous cells. In other words, p53 restoration was effective for adenocarcinoma but not for adenoma depletion (Feldser et al., 2010; Junttila et al., 2010). These results corroborate the evidence suggesting that high levels of oncogenic activity are necessary to trigger ARF–p53-mediated tumour suppression (Lin and Lowe, 2001). Also, it is known that a consistent low level expression of dominant oncogenes is sufficient for initiating tumorigenesis in the form of adenoma. Possibly, a low level of oncogene-induced DNA replication stress or other signalling cues might not suffice for p53 activation and subsequent induction of p21 expression. Consistent with this idea, enhancing the DNA damage response signalling by γ-irradiation promoted p21 expression across different tissues upon p53 restoration (Junttila et al., 2010). These lines of evidence suggest that a threshold of sensitivity to DNA damage response (DDR) appears to be one of the requirements for p53-induced cell cycle arrest or its apoptotic programme. Altogether, it can be conceived from the literature that p53 restoration effectively prevents tumour progression but is not capable of preventing tumour initiation.

### Mechanism of RUNX3-mediated prevention of pre-cancerous lesion and tumour progression

**Figure 1:**
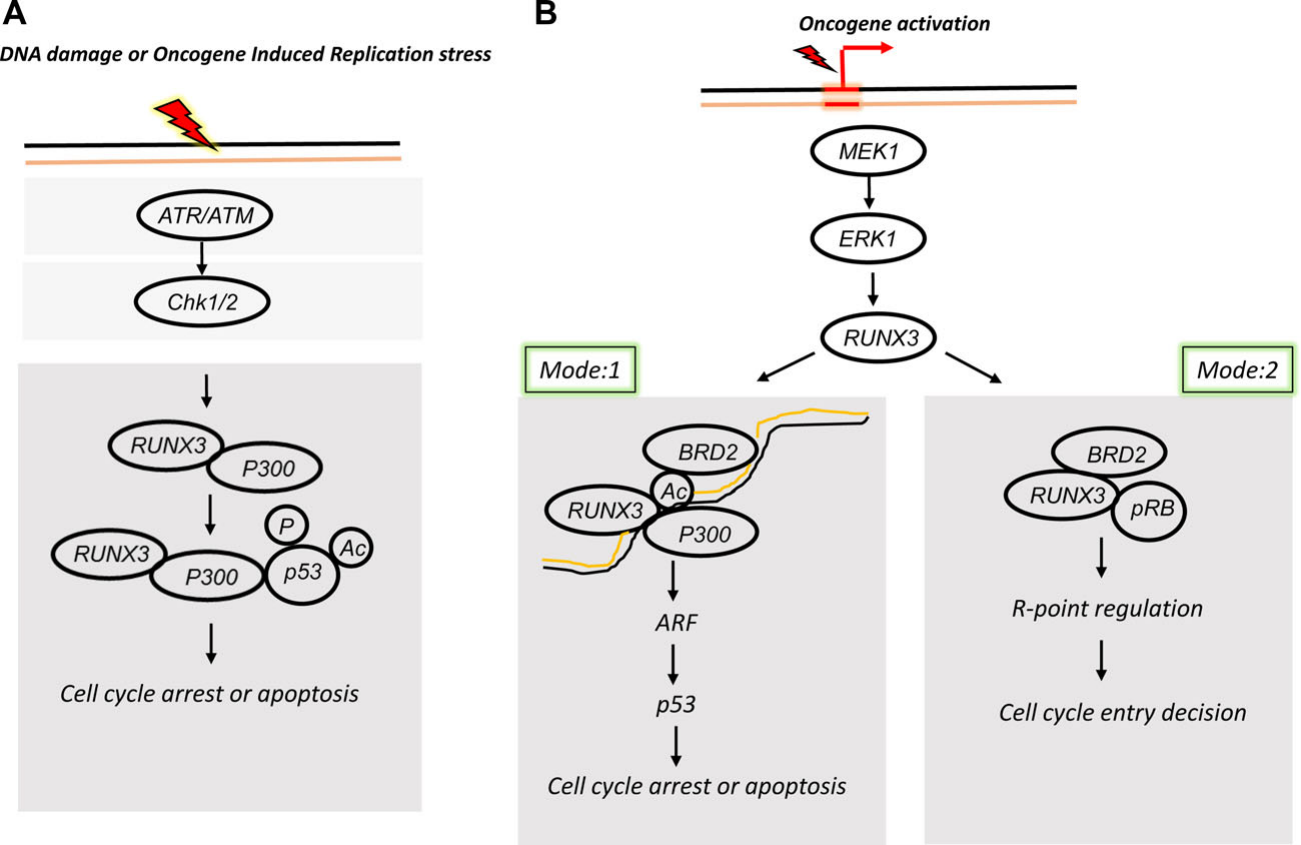
RUNX3 and p53 act independently and in concert to elicit their tumour suppressive function. (**A**) DNA damage or oncogene-induced replication stress induces ATR/ATM-mediated checkpoint activation. Activation of checkpoint machinery induces interaction between RUNX3 and p300, which in turn stabilizes phosphorylated and acetylated p53 to allow transcription of p53-dependent genes for cell cycle arrest or apoptosis. (**B**) On the other hand, oncogene activation induces mitogenic signalling by activation of downstream MEK1–ERK1 pathway. Mode 1: this signalling event allows the interaction between RUNX3–p300–BRD2 inducing transcription of ARF. ARF expression stabilizes p53 at protein level allowing transcription of p53 target genes. Mode 2: mitogenic signalling stimulates RUNX3–BRD2–pRB complex formation. This complex binds onto p21 promoter through RUNX-binding sites and induces p21 expression. Prolonged p21 expression inhibits further cell cycle progression.

An intrinsic relationship between RUNX and p53 stems from their ability to interact with each other in the presence of DNA damaging agents. RUNX3 interacts with p53 in the presence of adriamycin-induced DNA damage (Yamada et al., 2010). Adriamycin induces DNA double-strand break (DSB) by inhibiting Topoisomerase II, an enzyme required to release topological tension during DNA replication and transcription. In response to DNA damage, the interaction between RUNX3 and p53 enhances the phosphorylation of p53 at Serine 15 residue (Lee et al., 2017). This phosphorylation event stabilizes p53 and promotes apoptosis (Figure 1A). Consistently, RUNX3 deficiency reduces the transcription of p53-dependent target genes (Yamada et al., 2010). Overall the evidence suggests that the RUNX3-p53 complex triggers the apoptotic programme in cooperation with different factors and signalling events.

As stated above, it is apparent that p53 is not engaged in early stages but act at later stages of lung carcinogenesis. Therefore, it is important to consider the possibility that initiation of lung tumorigenesis might be suppressed by other tumour suppressor(s), whose loss might induce pre-cancerous lesions or adenomas. Earlier evidence suggests that RUNX3 is inactivated by hyper-methylation of RUNX3 promoter region and by hemizygous deletion in approximately 60% of primary gastric cancers (Li et al., 2002). *RUNX3^−/−^* knockout mice displayed hyperplasia of the stomach on the first day of the birth (Li et al., 2002) whereas *RUNX3-*heterozygous knockout induced adenomas across different tissues after a long latency (Chuang et al., 2013). Generally, *RUNX3* inactivation is frequently associated with human lung adenocarcinoma in a K-*Ras*-mutated background. Inactivation of Runx3 in lung was achieved by nasal infection of Adeno-Cre in the study reported earlier (Lee et al., 2013), which did not necessarily show that inactivation was achieved in epithelial cells. More recently, we expressed Cre recombinase specifically in Clara or AT2 cells and obtained essentially the same results (unpublished observations by Bae’s laboratory).

Earlier, inactivation of Runx2 in primary mouse embryo fibroblast (MEF) was shown to induce immortalization (Kilbey et al., 2007). Similar observation was made by inactivation of Runx3 (Lee et al., 2013). Then the complex relationship between RUNX, ARF and p53 has been addressed. By using oncogenic *Ras*-driven mouse model system, it has been shown that *Runx3* inactivation (*Runx3*^*f/f*^) alone induces lung adenoma (pre-cancerous lesion) and rapidly stimulates K-*Ras*-induced progression of adenocarcinoma of lung in mouse models (Lee et al., 2013). These results clearly demonstrate the existence of a defence mechanisms against early stages of lung tumorigenesis and that Runx3 plays a key protective role. Although K-*Ras*-induced lung adenocarcinoma development can proceed via multiple pathways, the frequent inactivation of RUNX3 by epigenetic silencing in K-*Ras*-induced human lung adenocarcinoma suggests that RUNX3 inactivation prior to K-*Ras* activation is a major contributing pathway.

Then, how does Runx3 defend against aberrant oncogene activation? Mitogenic signalling activates the GTPase activity of *Ras*, which decreases to the basal level soon after the signal is transduced to downstream kinase pathways. Oncogenic *Ras* is a constitutively active form whose activity is not downregulated. Therefore, heterozygous *Ras* mutation results in maintenance of a sustained increased level of *Ras* activity. To protect from oncogenic *Ras*-induced abnormal proliferation, cells should be able to sense the duration of the sustained *Ras* activity. For a long time, it remained unclear whether cells can indeed recognize aberrant persistent *Ras* activity. Recently, it has been demonstrated that Runx3 recognizes aberrant persistence of *Ras* activity by regulating the restriction (R)-point decision (Chi et al., 2017). The R-point is a critical event in which a mammalian cell makes the decision in response to mitogen stimulation (Pardee, 1974). After the R-point decision, depending on its differentiation stage, the cell either remains in early G1, retreats from the active cycle into G0, or advances into late G1. The postmitotic interval of G1, which lasts from mitosis to the R-point, is remarkably constant (3–4 h) in all tested cell lines (Zetterberg and Larsson, 1985). When *Ras* is activated by normal mitogenic stimulation, RUNX3 forms a complex with p300, pRB, and BRD2 (a relative of TAF250) in a MAPK activity-dependent manner; this complex transiently induces ARF, which in turn transiently stabilizes p53 (Figure 1B). Soon after the mitogenic surge, MAPK activity is reduced (Lee et al., 2013; Chi et al., 2017). In this situation, the pRB–RUNX3–BRD2 complex dissociates and ARF expression is repressed. Mitogen-stimulated transient activation of the ARF–p53 pathway does not affect the cell cycle because it occurs only 1–3 h after mitogenic stimulation and is then silenced at the G1/S checkpoint. When K-*Ras* is constitutively activated, the pRB–RUNX3–BRD2 complex is maintained and the expression of ARF and p53 continued until the G1/S checkpoint (Lee et al., 2013; Chi et al., 2017). These results show that cells can effectively defend against an endogenous level of *Ras* activity through RUNX3–ARF–p53 pathway, and that the pRB–RUNX3–BRD2 complex functions as a sensor for abnormal persistence of *Ras* activity. Furthermore, these results also provide a possible explanation as to why K-*Ras*-activated tumours recur so quickly: cells with K-*Ras* mutation are selected only if they occur in R-point-disrupted cells, some of which harbour epigenetically inactivated *RUNX3*. Suppression of K-*Ras* cannot restore the already silenced RUNX3, and therefore cannot recover the R-point. Hence, these RUNX3-silenced cells cannot defend against oncogene activation and might therefore be responsible for recurrence. Because K-*Ras* activation is the most frequently detected genetic alteration in human tumours, identification of therapeutic targets for K-*Ras* activated tumours and resistance mechanisms to K-*Ras* inhibition would be of enormous therapeutic relevance. In this regard, it is worth emphasizing that in multiple types of tumours, RUNX3 is frequently inactivated by epigenetic alterations, which could in theory be reversed. Therefore, RUNX3 represents a therapeutic target for diverse tumour types. Hence the function of RUNX3 in response to oncogenic stimuli appears at least one of the first line of defence mechanisms to eliminate cells that might undergo transformation. Collectively, the findings clarify a sequence of tumour suppressive events beginning from RUNX3 expression to p53 stabilization.

## Transcription-independent DNA repair function of RUNX and p53

### RUNX—role in DNA damage response and repair

Recently, a transcription-independent DNA repair function for RUNX1/3 was documented. *Runx1*/*Runx3* knockout mice showed bone marrow failure (BMF) and myeloproliferative disorder (Wang et al., 2014). These contradictory clinical manifestations are primarily observed in Fanconi anaemia (FA) patients. FA is a genetic disorder characterized by predisposition to various types of cancer including head and neck, breast, and ovarian cancers (Catucci et al., 2018). FA patient-derived cells are incapable of repairing a toxic lesion called inter-strand cross links (ICL) that arises when covalently attached DNA double strands prevent helicase-catalyzed DNA unwinding during DNA replication.

**Figure 2:**
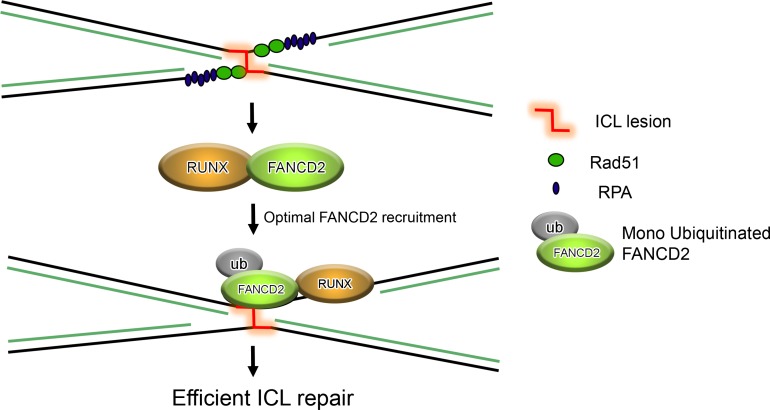
ICL lesion induces stalling of replication forks resulting in accumulation of Rad51 and RPA. Induction of ICL lesion results in RUNX and FANCD2 interaction, which is required for optimal loading of FANCD2 onto damage sites. The figure is adapted from Tay et al. (2018).

Investigation of the role of RUNX1/3 in the context of FA pathway revealed that RUNX1/3 is required for efficient ICL repair (Figure 2). Furthermore, RUNX1/3 knockout mice showed increased sensitivity and enhanced accumulation of γ-H2AX in the presence of ionizing radiation and mitomycin C (Wang et al., 2014). Prolonged accumulations of γ-H2AX in the absence of RUNX1/3 are signs of inefficient DNA repair activity or genome instability. Detailed molecular analysis revealed that RUNX1/3 loss significantly impaired FANCI/FANCD2 chromatin association and foci formation in the presence of ICL-induced DNA damage (Wang et al., 2014). FANCD2/FANCI are key players of ICL repair that promote unhooking of lesions in co-operation with nucleases such as XPF-ERCC1 and SLX4, thereby allowing the recruitment of homologous recombination (HR) machinery to complete error-free DNA repair (Klein Douwel et al., 2014; Zhang et al., 2015). Immunoprecipitation experiments have shown that in the presence of DNA damage, RUNX interacts with FA proteins FANCI/FANCD2 to facilitate their loading onto the sites of ICL (Figure 2). Of note, RUNX interaction with FA proteins is independent of its canonical transcription complex RUNX/CBFβ. Overall, these findings hint at a transcription-independent function for RUNX in maintaining genome stability.

Along the same lines, *in vitro* biochemical reconstitution experiments with intact nuclear extracts revealed that in the presence of induced DNA damage, RUNX protein complexes bound single stranded DNA (ssDNA) and splayed arm DNA with greater affinity compared to double stranded DNA (Tay et al., 2018). Of importance, RUNX interactions with these DNA replication/repair intermediates are independent of its affinity for the consensus motif involved in RUNX-mediated transcription. Moreover, inhibition of DDR pathways ATR and PARP1 attenuated the affinity of RUNX for ssDNA and splayed arm DNA. ATR kinase is activated in the presence of DNA replication stress when extensive ssDNA regions are coated with ssDNA binding protein RPA together with its newly discovered partner ETAA1 (Haahr et al., 2016). In parallel, PARP1 is an enzyme that poly-ADP ribosylates a wide range of proteins, regulating the choice of DNA repair pathways (Ray Chaudhuri and Nussenzweig, 2017). Collectively, these findings point to an interplay between RUNX and DNA replication/repair intermediates in the presence of DNA damage. Future investigation on RUNX in the DNA repair context is necessary to further clarify the role of RUNX and its protein complexes at the site of repair.

Furthermore, we cannot rule out the possible involvement of RUNX proteins in combination with FA proteins in R-loops regulation. R-loops are RNA:DNA hybrids, a co-transcriptional product that has a physiological function but aberrant accumulation of R-loops poses a threat to genome stability (Aguilera and Garcia-Muse, 2012). FANCD2 has been shown to prevent R-loop accumulation, thereby protecting genome integrity (Garcia-Rubio et al., 2015). Recent Chip-Sequencing analysis of FANCD2 suggested that FANCD2 predominantly associates with regions of active transcription and common fragile sites (Okamoto et al., 2018), hinting at a possibility of RUNX-FANCD2 cooperation on those sites. However, these ideas warrant further investigation. It is worth mentioning that besides ICL repair, FA pathway proteins are implicated in genome maintenance during unperturbed DNA replication and in the presence of replication stress (Madireddy et al., 2016; Kolinjivadi et al., 2017). Furthermore, FANCD2 has been shown to be required for replication fork stability in the presence of DNA damage induced by oncogene, HU (Hydroxyurea), aphidicolin, acetaldehyde, and malonil aldehydes (Langevin et al., 2011; Tian et al., 2017). Of note, FANCD2 is indispensable for DNA replication fork protection and restart in *BRCA2*-deficient cancer cells (Kais et al., 2016). Further, FANCD2 enhances error-prone alternative-End Joining (alt-EJ) at damage sites by promoting optimal loading of Pol θ, a polymerase required for error-prone repair (Kais et al., 2016). Hence there exists a synthetic lethal relationship between *FANCD2* and *BRCA2*. In this context, it is important to address the role of RUNX proteins in BRCA2-deficient and proficient backgrounds. Investigating the complex relationship between RUNX, FANCD2/FANCI and BRCA2 will be an interesting area for future research.

### p53—non-transcriptional genome maintenance function

Recently, p53 was shown to play a direct role in DNA replication fork restart (Roy et al., 2018). Genetic evidence from p53 mutant mice hints at a transcription-independent function for p53. For instance, although the human p53 R175P and its corresponding murine orthologue p53 R175P mutant proteins lose their transcriptional activity, *p53 R175P* mutant mice retain their potent tumour suppressive activity (Liu et al., 2004). On the other hand, mutations in acetylation region of p53 impair the ability of p53 to transactivate transcription but the acetylation-mutant mouse models still showed delayed neoplastic onset, when compared to p53-null mice (Li et al., 2012; Zhu et al., 2015). These findings suggest that p53 possesses a non-transcriptional activity that protects cells from genome instability. Moreover, p53 is activated by stalled DNA replication forks that potentially arise due to strong oncogene-induced DNA replication stress (Gottifredi et al., 2001; Kumari et al., 2004). Also, it has been shown that in the presence of induced DNA replication stress, p53 interacts with BLM helicase to suppress unscheduled HR during S phase (Janz and Wiesmuller, 2002; Saintigny and Lopez, 2002; Bertrand et al., 2004). Furthermore, an independent study showed that p53 interacts with DNA POLi, a polymerase implicated in DNA damage tolerance (Hampp et al., 2016). This interaction is required to regulate replication fork progression in the presence of replication stress. Very recently, using elegant techniques such as DNA fibre assay, isolation of proteins enriched on nascent DNA (iPOND), and *in situ* analysis of protein interactions at DNA replication forks (SIRF), Roy et al. (2018) demonstrated that p53 bound onto stalled DNA replication forks and promoted replication fork restart. Mechanistically, they found that in the presence of DNA replication fork stalling agents, p53 promotes MLL3 (a methyl transferase)-mediated chromatin remodelling. This chromatin remodelling event attracts MRE11 nuclease to restart DNA replication forks in a timely manner to prevent replication fork collapse (Roy et al., 2018). Further, they showed that loss of p53 enhances the association of error-prone repair factors Rad52 and Pol θ leading to mutations and genome instability. Overall, these findings suggest a replication fork restart function for p53 in the presence of DNA replication stress—a conceivable explanation as to why loss of p53 leads to genomic instability. With a spectrum of different functions, it is unclear how p53 mediates MLL3-mediated chromatin remodelling at stalled replication forks; how does p53 restrict the binding of Rad52 and Pol θ onto stalled replication forks? Targeted investigation of p53 activities at the replication fork might shed light on the transcription-independent role of p53 in DNA repair.

## Concluding remarks

Both p53 and RUNX genes are evolutionarily very old. They are present in the genome of amoeboid holozoan *Capsaspora owczarzaki*, a unicellular organism considered to be the precursor to metazoans (Sebe-Pedros et al., 2011). During evolution, as organisms acquired more and more complex functions, these two family of genes adapted to function in varied biological processes. p53 is well recognized as a tumour suppressor. Of all the functions associated with p53, it is likely that its primordial function is to monitor genome stability and maintain cellular homoeostasis (Junttila and Evan, 2009; Lu et al., 2009). RUNX genes are less characterized but considering their abilities to defend against stress and external stimuli, the primordial function of RUNX may also be to protect the genome of the unicellular organism. Here, these two families of genes show different modes of action as tumour suppressors. The most striking observation is that the RUNX proteins protect cells at the early stage of cancer. For example, RUNX3 prevents adenoma formation (Chi et al., 2017). In contrast, as discussed above, p53 functions at later cancer stages to prevent adenocarcinoma (tumour progression). RUNX protein functions in DNA repair pathways and at the R-point of cell cycle seem to explain their involvement in the early stage of carcinogenesis (Wang et al., 2014; Chi et al., 2017). On the other hand, p53’s conspicuous role is to eliminate transforming cells from the body to protect the well-being of the organism, as highlighted by the frequent inactivation of p53 in many cancer types. It has been argued that the frequency of RUNX3 mutation pales when compared to that of p53 mutation in cancer. Yet, epigenetic silencing of RUNX3 is extremely common in cancer and often unappreciated. We propose a two-step defence mechanism for RUNX and p53, the first barrier by RUNX family gene to prevent adenoma formation and the second barrier by p53 to prevent cells from transforming to adenocarcinoma. An important point to consider is how oncogenes behave when RUNX3 or p53 is inactivated. It has been shown that activated oncogene stimulates RUNX3–BRD2 interaction which in turn activates Arf and p53 (Lee et al., 2013; Chi et al., 2017). In the absence of RUNX3, p53 is not activated. Therefore, cells with activated K-*Ras* might gain a proliferative advantage. Moreover, RUNX3 loss results in deregulation of the R-point. Under such conditions, oncogenic stimuli are not counteracted by RUNX3/pRB complex. As a consequence, cells enter prematurely into S phase, resulting in unscheduled commitment to cell cycle.
